# Photodynamic therapy and peri-implant diseases: a systematic review and meta-analysis

**DOI:** 10.3389/froh.2025.1614982

**Published:** 2025-07-09

**Authors:** Yumeng Yan, Roberto Rotundo, Jeanie Suvan, Marco Orlandi, Alessandro Poma, Francesco D’Aiuto

**Affiliations:** ^1^ Periodontology Unit, UCL Eastman Dental Institute, London, United Kingdom; ^2^ Periodontology Unit, University Vita-Salute San Raffaele, Milan, Italy; ^3^ Oral Sciences, University of Glasgow Dental School, School of Medicine, Dentistry and Nursing, College of Medical, Veterinary and Life Sciences, University of Glasgow, Glasgow, United Kingdom; ^4^ Biomaterials and Tissue Engineering, UCL Eastman Dental Institute, London, United Kingdom

**Keywords:** photodynamic therapy, antimicrobial, peri-implantitis, peri-implant disease, peri-implant mucositis

## Abstract

**Aim:**

This systematic review aimed to evaluate the antimicrobial efficacy of photodynamic therapy (PDT) in treating peri-implant diseases when combined with mechanical debridement (MD) compared with mechanical debridement alone.

**Methods:**

A systematic review was completed according to PRISMA guidelines. The following databases, Cochrane Central Register for Controlled Trials (CENTRAL), Medline, Embase, Dentistry & Oral Sciences Source, Scopus, LILACS, and China Online, were searched based on the search strategies and hand search without language limitation until 15 June 2024. Only randomised controlled trials were included, assessing the efficacy of PDT used in combination with either surgical or non-surgical MD, compared with MD alone in participants with peri-implant diseases. Risk of bias for randomised controlled trials was assessed according to the recommendation of the Cochrane Reviewers' Handbook using the revised Cochrane tool. All outcomes were evaluated using the Grading of Recommendations Assessment, Development, and Evaluation (GRADE) approach.

**Results:**

A total of 26 studies were included in this study, of which 6 were categorised as low risk of bias, 9 were with some concern, and 11 were at high risk of bias. Nineteen studies were included in the quantitative analysis. At 3 months, PDT combined with non-surgical MD significantly reduced probing pocket depth (PPD) in peri-implant mucositis (−0.95 mm, 95% CI: −1.76 to −0.14) and peri-implantitis (−0.86 mm, 95% CI: −1.21 to −0.51) compared with MD alone. At 6 months, PPD reductions in peri-implantitis remained significant with non-surgical MD + PDT (−0.83 mm, 95% CI: −1.62 to −0.04) and surgical MD + PDT (−0.56 mm, 95% CI: −0.85 to −0.27). Non-surgical MD + PDT also reduced bleeding on probing (BoP) (−11.65% at 3 months, −6.76% at 6 months) and crestal bone loss (CBL) (−0.24 mm at 3 months, −0.28 mm at 6 months).

**Conclusion:**

PDT enhances antimicrobial efficacy in peri-implant disease treatment, significantly improving PPD, CBL, and BoP when combined with MD. However, due to the overall moderate-to-low certainty of the evidence and some concerns regarding risk of bias in the included studies, these findings should be interpreted with caution. Further high-quality, well-designed randomised controlled trials are warranted to confirm these effects and optimise treatment protocols.

**Systematic Review Registration:**

PROSPERO CRD42021262889.

## 1 Introduction

Peri-implant diseases, consisting of peri-implant mucositis and peri-implantitis, are increasingly prevalent due to the widespread use of dental implants for the replacement of missing teeth (1). Peri-implant mucositis is defined as an inflammatory disease that only involves soft tissue inflammation around dental implants, while peri-implantitis is characterised by inflammation in the mucosa as well as progressive loss of supporting bone tissue (2).

Aetiological evidence supports bacterial accumulation as a key determinant of the onset and progression of peri-implant diseases. However, the association between a specific bacterial cluster and peri-implantitis remains unclear. Bacteria commonly linked to periodontitis, such as *Bacteroides*, *Campylobacter*, *Eubacterium*, *Fusobacterium*, and *Treponema species*, are frequently identified in peri-implantitis (3, 4). Higher proportions of the genera *Fusobacterium* and *Bacteroides* have been identified in the peri-implant samples who are smokers when compared with never smokers (5). In addition, less common oral species, including *staphylococci*, *enteric bacteria*, and *yeasts*, have been recovered from failing implants, highlighting the increased complexity of the microbiota in peri-implantitis (6).

The plaque biofilm can trigger inflammation around dental implants and would result in soft and hard tissue destruction if left untreated (7). Treatment strategies of peri-implant diseases primarily focus on the decontamination of the implant surface and the reduction of bacteria in peri-implant tissues. Both non-surgical and surgical treatments for peri-implantitis are considered therapeutic regimens to manage bacterial biofilm (8). Studies have reported that mechanical debridement (MD) alone has limited effectiveness in the non-surgical treatment of peri-implantitis (9). Moreover, decontamination of a threaded metal surface, as that of dental implants, is more challenging than root dental surfaces; hence, surgical treatment facilitates access (10). Bacterial biofilms are intrinsically resistant to the metabolic activity required for standard antibiotic regimens to be effective. Therefore, it is imperative to develop highly effective alternative antimicrobial strategies to address and eliminate chronic or recurrent infections associated with biofilms. Ideally, these new treatment approaches should with killing mechanisms that minimise or prevent the development of microbial resistance (11). As a promising alternative to antibiotics, photodynamic therapy (PDT) is a non-invasive treatment using molecular energy produced by specific wavelength laser lights and photosensitive medication and resulting in the production of reactive oxygen species (ROS) with high chemical reactivity (12). PDT has a history in the management of periodontitis, where it has been used as an adjunctive antimicrobial approach to enhance the effects of conventional therapy (13). More recently, it has been used to treat peri-implant diseases. Previous published evidence, however, showed contradicting results on the effectiveness of PDT in managing peri-implant diseases, and comparisons between different PDT treatment protocols have not been adequately explored (13–15). Therefore, this systematic review and meta-analysis was designed to perform a comprehensive appraisal of all the evidence to date reporting on whether PDT combined with surgical or non-surgical MD improves clinical outcomes compared with mechanical debridement alone in adults with peri-implant mucositis or peri-implantitis, with a minimum follow-up of 3 months.

## 2 Methods

### 2.1 Protocol

A rigorous review protocol was developed and implemented in alignment with the Preferred Reporting Items for Systematic review and Meta-Analysis (PRISMA) 2020 guidelines (16) and registered on PROSPERO (reference no: CRD42021262889).

### 2.2 Eligibility criteria

#### 2.2.1 Focused question

The focused research question was: “Can antimicrobial photodynamic therapy combined with surgical or non-surgical MD improve the clinical outcomes in peri-implant mucositis or peri-implantitis compared with MD alone, with a minimum of 3 months follow-up?”.

#### 2.2.2 PICOS

#### The PICOS framework was applied as follows:

P (Population)—adult population diagnosed with peri-implant mucositis or peri-implantitis.

I (Intervention)—photodynamic therapy combined with surgical or non-surgical MD.

C (Comparison)—surgical or non-surgical MD alone.

O (Outcome)—primary outcome: probing pocket depth (PPD) reduction. Secondary outcomes: improvement of (1) clinical attachment level (CAL); (2) bleeding on probing (BoP); (3) gingival index (GI); (4) plaque index (PI); (5) sulcus bleeding index (SBI); (6) modified sulcus bleeding index (mSBI); (7) crestal bone loss (CBL); (8) plaque score (PS); (9) modified plaque index (mPI); and (10) modified gingival index (mGI) reduction.

S (study): Only randomised controlled trials (RCT) with at least a 3-month follow-up were included.

### 2.3 Search strategy

Broad and inclusive electronic search strategies were applied to include citations until 15 June 2024. The following electronic databases were searched without language limitation using medical subject headings and free-text terms: Cochrane Central Register for Controlled Trials (CENTRAL), Medline, Embase, Web of Science, Dentistry & Oral Sciences Source, Scopus, LILACS, and China Online. The following journals were searched by hand since 2005: Journal of Periodontology, Journal of Clinical Periodontology, Clinical Oral Implants Research, Journal of Oral Implantology, and Clinical Implant Dentistry and Related Research. Two trial registers, namely, ClinicalTrials.gov and WHO ICTRP, were searched. The SIGLE database was searched for grey literature (Appendix Table A1). Search results retrieved from the electronic searches were imported into a reference management software, and duplicates were removed (EndNote, version 20).

### 2.4 Study selection

Titles and abstracts were screened independently by two reviewers (YY and RR). Full-text articles were obtained for studies where there was insufficient information in the title and abstract to make a clear decision. The full reports were assessed independently, in duplicate, by the same two reviewers to establish eligibility for inclusion. If manuscripts were lacking information necessary for analysis, authors were contacted to retrieve missing data. Disagreement was resolved by discussion, and if necessary, a third reviewer was consulted (MO).

### 2.5 Risk of bias evaluation of selected studies

Data were extracted into evidence tables. The extracted data included study characteristics, peri-implant status, definition of peri-implant diseases, mean age, smoking habit, intervention, photosensitiser type, laser properties, irradiation time, and conclusion. Quality assessment and risk of bias for randomised controlled trials were assessed according to the recommendation of the Cochrane Reviewers' Handbook using the revised Cochrane tool (RoB 2) (17). A qualitative review with descriptive analysis was performed to determine the quality of data, checking for the level of risk of bias for the studies and selecting studies suitable for inclusion in quantitative analyses.

### 2.6 Data synthesis and grading

All data retrieved that could be used in quantitative analyses were analysed using STATA statistical software 18.0MP Parallel Edition (StataCorp LLC, College Station, TX, USA), including PPD reduction and improvement of CAL, BoP, GI, PI, SBI, mSBI, CBL, PS, mPI, and mGI at a minimum of 3-month follow-up. Mean differences were calculated with a 95% confidence interval (CI). The chi-square-based *Q*-statistic method and *I*^2^ measurements were employed to assess the heterogeneity. The pooled estimates were calculated using random-effects models because of the expected heterogeneity between studies. The pooled effect was considered significant if *p* < 0.05. Egger's test (18) was generated to assess whether small studies generate larger treatment effects (19). All outcomes were then evaluated using the Grading of Recommendations Assessment, Development, and Evaluation (GRADE) approach (20).

### 2.7 Subgroup analyses

Subgroup analyses were generated to explore the sources of heterogeneity for the studies based on the following factors:

1) Smoking habit: smokers and non-smokers were analysed separately.
2) Case definition: studies were grouped by case definition and analysed separately.
3) Treatment protocol of PDT: PDT with different kinds of photosensitisers were analysed.

## 3 Results

### 3.1 Selection and characteristics of included studies

**Figure 1:**
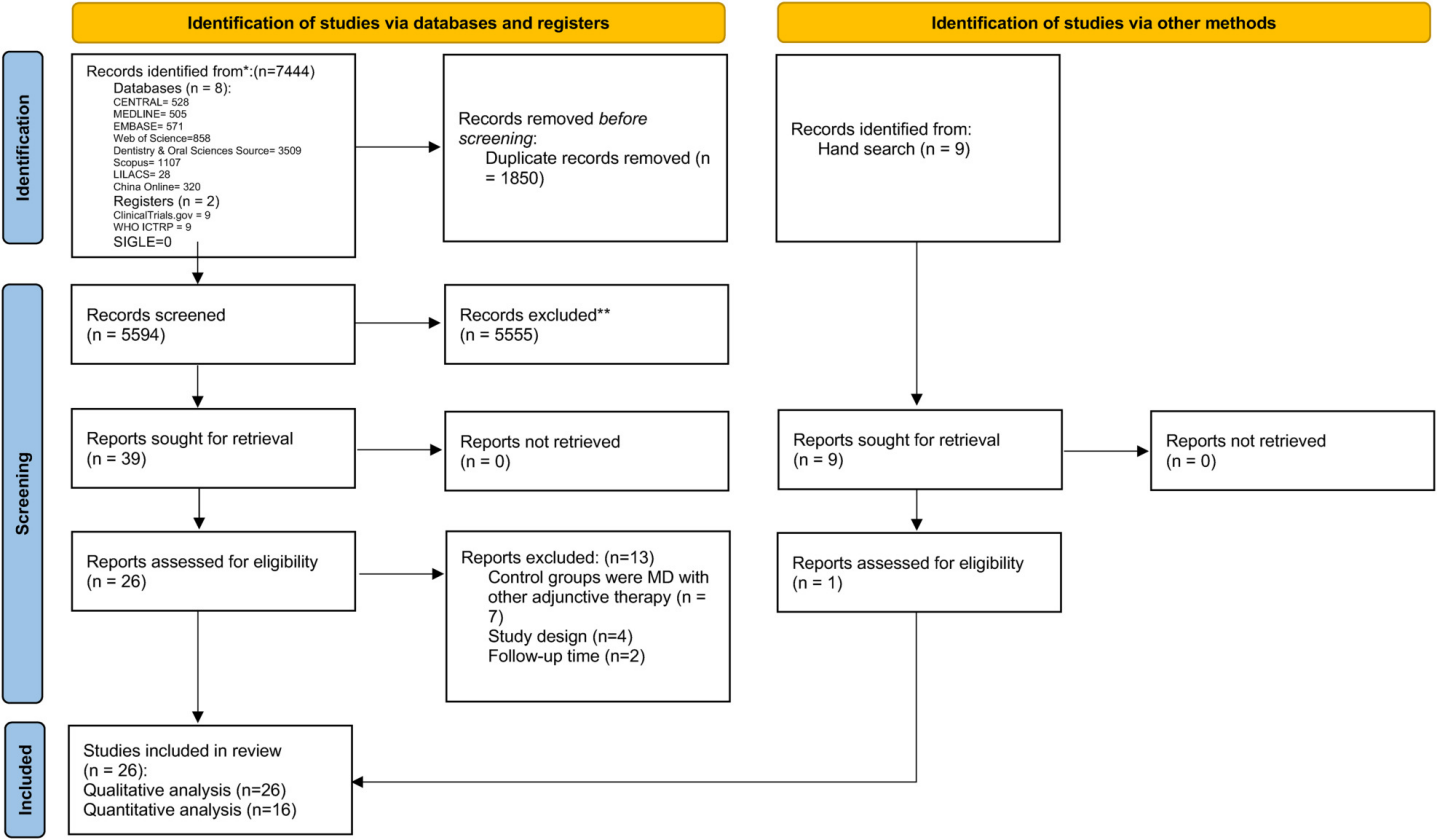
PRISMA flowchart of the study selection process, each stage of identifying and selecting studies.

The electronic search identified 7,444 hits. After removing 1,846 duplicates and incorporating results from the hand search, the title and abstract screening identified 39 articles eligible for full-text assessment. Two studies were excluded due to a lack of sufficient follow-up time (21, 22), seven studies used control groups with other adjunctive therapy (23–29), and four were non-RCTs (30–33). A total of 26 articles were eligible for qualitative analysis (Figure 1 and Table 1). PPD was assessed in 25 studies, BoP in 14 studies, CBL in 10 studies, PI in 12 studies, mPI in 4 studies, SBI in 2 studies, mSBI in 2 studies, mGI in 1 study, GI in 1 study, PS in 4 studies, CAL in 3 studies, and BI in 2 studies. After assessment of available data, seven studies were not eligible for meta-analysis due to a lack of mean or standard deviation (34–40). Another study was not included in the quantitative analysis due to the limited number of studies with a 12-month follow-up time point (41). Two studies were excluded from quantitative analysis due to merged peri-implantitis and peri-implant mucositis patients in one group (12, 42). A total of 16 randomised controlled trials with 1,205 participants (43–58) were deemed eligible for quantitative analysis.

**Table 1:** Participants’ characteristics and study design of included studies.

Author, year of publication, and country	Peri-implant status	Definition of peri-implant diseases	Mean age [years (range) or ±(SD)]	Smoking habit	Method	Photosensitiser	Laser type, wavelengths, power, and irradiation time	Conclusion	Conflict of interest
Li et al., 2013, China (43)	Peri-implantitis	SBI ≥1, PPD ≥4 mm, or suppuration, x-ray shows bone loss around the neck of the implant	47 (23–69)	NA	Control: *n* = 15, full mouth scaling and root planning, MD Test: *n* = 15 full mouth scaling and root planning, MD with adjunctive PDT	Toluidine blue (100 g/ml)	Diode laser, 635 nm; 750 mW; irradiation time: NA	Adjunctive PDT with SRP is more effective than SRP alone	Not reported
Esposito et al., 2013, Sweden (41)	Peri-implantitis	At least one osseointegrated implant that lost at least an average of 3 mm of peri-implant bone from the baseline	Test: 58.0 (36–79) Control: 60.2 (25–80)	Non-smoker, moderate smoker (10 cigarettes per day) and heavy smoker (>10 cigarettes per day)	Control: *n* = 40, non-surgical or surgical debridement based on marginal bone loss Test: *n* = 40, non-surgical or surgical debridement based on marginal bone loss with adjunctive PDT	Toluidine blue O (0.1 mg/ml)	Wavelengths; power: NA; 80 s	Adjunctive PDT with mechanical cleaning of implants affected by peri-implantitis did not improve any clinical outcomes when compared with mechanical cleaning alone up to 1 year after treatment	The manufacturer of the PDT device partially supported this trial; however, when results became available, the manufacturer did not honour the financial agreement
Bombeccari et al., 2013, Italy (44)	Peri-implantitis	PPD ≥5 mm, with the presence of BoP and/or inflammatory exudation. Radiographic signs of progressive bone loss (bone loss three threads) around the dental implant for at least 12 months	46 (33–64)	Exclude smoking >10 cigarettes per day	Control: *n* = 20, surgical therapy (mucoperiosteal flap surgery with scaling on implant surfaces and debridement of granulation tissue) Test: *n* = 20, surgical therapy with adjunctive PDT	Toluidine blue O (TBO 100 mg/ml)	Diode laser, 810 nm; 1 W; 20 s	Adjunctive PDT was not associated with decontaminating anaerobic bacteria on a rough titanium implant. However, PDT appears to significantly reduce clinical signs of peri-implant inflammation, leading to lower bleeding scores and inflammatory exudates relative to the conventional approach	Nothing to disclose
Nicolae et al., 2015, Romania (40)	Peri-implantitis	PPD between 4 and 6 mm	Test: 40.8 ± 8.3 Control: 38.4 ± 9.6	Non-smokers	Control: *n* = 22, full mouth scaling and root planning, MD Test: *n* = 22, full mouth scaling and root planning, MD with adjunctive PDT	Tolonium chloride (0.01 mg/ml)	Doide laser, 635 nm; power: NA; 60 s	Adjunctive PDT with SRP is more effective than SRP alone	Not reported
Romeo et al., 2016, Italy (37)	Peri-implantitis	Pl ≥40% and at least one implant site with the following characteristics: PPD ≥4 mm, BoP, and presence of suppuration.	NA (34–68)	Exclude smoking >10 cigarettes per day	Control: *n* = 20, air polishing and implant debridement Test: *n* = 20, air polishing and implant debridement with adjunctive PDT	HELBO Blue (a liquid containing methylene blue) (10 mg/ml)	Diode laser, 670 nm; 75 mW/cm^2^; 60 s	PDT is a reliable adjunctive treatment to conventional therapy.	Nothing to disclose
Abduljabbar, 2016, Saudi Arabia (42)	Peri-implant diseases	Peri-implant disease: peri-implant BoP and PPD ≥4 mm ≥30% of sites	Test: 50.6 ± 1.4 Control: 51.4 ± 0.6	Non-smokers	Control: *n* = 30, patients underwent MD alone Test: *n* = 30, patients underwent MD with aPDT	Phenothiazine chloride	Diode laser, 660 nm;100 mW; 120 s	In the short term, MD with aPDT is more effective in the treatment of peri-implant inflammation compared with MD alone in prediabetic patients	Nothing to disclose
Karimi et al., 2016, Iran (12)	Peri-implant mucositis and peri-implantitis	Peri-implant mucositis: presence of BoP, soft tissue redness, PPD <5 mm, no peri-implant bone loss in radiographs Peri-implantitis: vertical, or saucer-shaped peri-implant bone loss in parallel periapical radiographs, compared with control x-ray obtained after prosthesis delivery, in addition to exhibiting at least two of the following clinical signs: BoP, suppuration and fistula, mucosal swelling and redness, PPD >5 mm, and mucosal recession	52.8 ± 7.33	NA	In each patient, one implant randomly served as control implant and the other served as test implant. The control implants were treated with closed surface scaling only and the test implants received additionally PDT	Toluidine blue 0.01%	Diode laser, 630 nm; 2,000 mW/cm^2^; 20 s	Adjunctive PDT with MD is more effective than MD alone	Nothing to disclose
Javed et al., 2016 (35)	Peri-implant diseases	BoP in at least 30% of sites and probing depth of at least 4 mm	Smokers: Test = 40.5 (28–54) Control = 40.5 (28–54) Non-smokers: Test = 41.6 (26–55) Control = 40.2 (28–51)	Both smokers and non-smokers	Control: smokers, *n* = 43; non-smokers, *n* = 42; MD Test: smokers, *n* = 41; non-smokers, *n* = 40; MD with aPDT	NA	NA	In the short term, MD with aPDT is more effective in reducing peri-implant probing depth than MD alone in smokers and non-smokers. However, in the long term, outcomes of MD either with or without aPDT are comparable among smokers and non-smokers	Nothing to disclose
Mehr et al., 2017, Iran (36)	Peri-implant mucositis	PPD: 3–6 mm, BoP, no soft tissue recession with or without minimum bone resorption ≤2 mm in periapical radiography	57 (29–70)	Non-smokers	Control: *n* = 25, ultrasonic scaling Test: *n* = 25, ultrasonic scaling with adjunctive PDT	Toluidine blue	Diode laser, 638 nm; 150 mW; 10 s	Adjunctive PDT has a significant effect in resolving BOP levels but had no additive effect in reducing PD in peri-implant mucositis	Nothing to disclose
Al-Sowygh, 2017, Saudi Arabia (34)	Peri-implant mucositis	BoP and PPD of at least 4 mm in at least 30% of sites	Test: 42.5 ± 6.5 Control: 46.8 ± 5.7	Smokeless-tobacco product users	Control: *n* = 24, mechanical curettage Test: *n* = 25, mechanical curettage + PDT	NA	NA	Among patients with peri-implant mucositis, MC with aPDT is more effective in reducing peri-implant inflammation in smokeless-tobacco product users as compared with mechanical curettage alone	Nothing to disclose
Wang et al., 2017, China (45)	Peri-implantitis	PPD 4–6 mm and BoP(+), x-ray showed low density of implant neck	43 (24–65)	Non-smokers	Control: *n* = 20, full mouth scaling and root planning, MD Test: *n* = 20, full mouth scaling and root planning, MD with adjunctive PDT	Toluidine blue 100 μg/ml	Diode laser, 635 nm; 750 mW; 60 s	Adjunctive PDT with MD is more effective than MD alone	Not reported
Javed et al., 2017, country: NA (46)	Peri-implant mucositis	PPD of at least 4 mm at least east 30% of sites	Test: 50.6 ± 0.8 Control: 52.2 ± 0.5	Smokers	Control: *n* = 28, mechanical curettage Test: *n* = 26, mechanical curettage with adjunctive PDT	HELBO Blue (a liquid containing methylene blue) (10 mg/ml)	Diode laser, 660 nm; 100 mW; 10 s	Adjunctive PDT with MD is more effective than MD alone	Nothing to disclose
Al Rifaiy et al., 2018, Saudi Arabia (48)	Peri-implant mucositis	NA	Test: 33.6 ± 2.8 Control: 35.4 ± 2.1	Vaping	Control: *n* = 20, MD Test: *n* = 18, MD with adjunctive PDT	Methylene blue, 0.005%	Diode laser, 670 nm; 150 mW; irradiation time: NA	Antimicrobial PDT is more effective compared with MD alone in the treatment of p-iM in individuals vaping e-cigs	Nothing to disclose
Albaker et al., 2018, Saudi Arabia (55)	Peri-implantitis	A minimum of one implant with peri-implant marginal bone loss ≥2 mm comparison of the bone level 1 year following implant reconstruction with the bone level at screening, or CBL ≥3 mm on x-ray, in combination with PPD ≥5 mm and with bleeding or suppuration on probing	Test: 58.4 ± 8 Control: 61.5 ± 9.9	Both smokers and non-smokers	Control: *n* = 28, mechanical curettage Test: *n* = 26, mechanical curettage with adjunctive PDT	Methylene blue, 0.005%	Diode laser, 670 nm; 150 mW; 60 s	MD with adjunctive PDT does not provide additional benefit in improving clinical and radiographic peri-implant parameters than MD alone	Nothing to disclose
Liu et al., 2018, China (47)	Peri-implantitis	PPD ≥5 mm around the implant, mSBI ≥1 or the site of pus ≥1, progressive bone resorption around implant	51.69 ± 9.33	Non-smokers	Control: *n* = 13, ultrasonic scaling and scaling and root planning Test 1: *n* = 13, ultrasonic scaling and scaling and root planning with adjunctive PDT Test 2: *n* = 13, ultrasonic scaling and scaling and root planning with minocycline hydrochloride ointment	Toluidine blue, 100 mg/L	Diode laser, 635 nm; 750 mW; 60 s	MD with adjunctive PDT can improve the clinical parameters and reduce the level of inflammatory cytokines in peri-implant crevicular fluid	Not reported
Wang et al., 2019, China (50)	Peri-implantitis	Soft tissue around the implant showing obvious inflammatory symptoms, bone loss by x-ray, probable haemorrhage and suppuration, at least one implant site with PPD ≥6 mm, PLI around the implant ≥2 points, visible BoP, with a SBI ≥2 points, CAL ≤3 mm	Test: 44.1 ± 9.8 Control: 42.6 ± 13	NA	Control: *n* = 65, full mouth cleansing, pocket cleansing and subgingival sandblast Test: *n* = 66, full mouth cleansing, pocket cleansing and subgingival sandblast with adjunctive PDT	Toluidine blue, 10 mg/ml	Diode laser, 635 nm; 750 mW; 10 s	Adjunctive PDT with MD is more effective in improving clinical parameters than MD alone	Nothing to disclose
Alqahtani et al., 2019, Saudi Arabia (49)	Peri-implantitis	PPD of ≥4 mm, and CBL ≥3 mm	Smokers: 52.3 ± 2.2 Never smokers: 54.2 ± 2.2	Separated patients into smokers, non-smokers, and waterpipe users	Non-smokers: Control: *n* = 16, ultrasonic scaling and hand curettes removal. Test: *n* = 16, ultrasonic scaling and hand curettes removal with adjunctive PDT. Smokers: Control: *n* = 17, ultrasonic scaling and hand curettes removal. Test: *n* = 17, ultrasonic scaling and hand curettes removal with adjunctive PDT	Methylene blue, 0.005%	Diode laser, 660 nm; 150 mW; 60 s	Adjunctive PDT with PD is more effective than MD alone	Not reported
Ahmed et al., 2020, Saudi Arabia (51)	Peri-implantitis	Mild probing leading to bleeding and/or suppuration; CAL ≤3 mm, PPD ≥6 mm, alveolar bone loss ≥3 mm	Control: 50.7 ± 5.9 Test 1: 48.9 ± 4.5 Test 2: 51.4 ± 4.4	Non-smokers	Control: *n* = 20, MD Test 1: *n* = 20, PDT + MD Test 2: *n* = 20, AGT (antibiotic gel therapy) + MD	Phenothiazine chloride	Diode laser, 660 nm; 150 mW; 10 s/site	Treatment of peri-implantitis using aPDT among Type 2 diabetes patients improved the clinical, radiographic, and immunological peri-implant parameters	Not reported
Al Deeb et al., 2020, Saudi Arabia (58)	Peri-implantitis	BoP and/or suppuration, PPD ≥6 mm, CBL ≥3 mm	Control: 49.2 ± 0.13 Test 1:52.6 ± 0.9 Test 2: 53.8 ± 0.7	Smokers	Control: *n* = 15, MD with titanium curettes and polishing using rubber cups and paste Test 1: *n* = 15, MD with titanium curettes and polishing using rubber cups and paste with adjunctive PDT Test 2: *n* = 15, MD with titanium curettes and polishing using rubber cups and paste with systemic azithromycin	HELBO Blue (a liquid containing methylene blue)	Diode laser, 660 nm; 100 mW; 10 s	Adjunctive PDT reduced the clinical peri-implant inflammation but no significant change in bone biomarkers	Not reported
Labban et al., 2021, Saudi Arabia (52)	Peri-implantitis	2018 new classification scheme for periodontal and peri-implant diseases and conditions	Test: 50.4 ± 9.3 Control: 47.8 ± 7.2	Non-smokers	Control: *n* = 18, MD Test: *n* = 18, MD with PDT	Indocyanine green solution	Diode laser, 810 nm; 200 mW; 10 s/site	Multiple application of indocyanine green-mediated photodynamic therapy resulted in improved clinical and microbial parameters among Type 2 diabetes mellitus subjects in the treatment of peri-implantitis	Not reported
Al-Askar et al., 2022, Saudi Arabia (53)	Peri-implantitis	2018 new classification scheme for periodontal and peri-implant diseases and conditions	Control: 62.8 ± 2.5 Test 1: 65.2 ± 1.3 Test 2: 63.2 ± 2.8	Non-smokers	Control: *n* = 16, MD Test 1: *n* = 16, MD and adjunctive PDT Test 2: *n* = 17, MD and photobiomodulation	Methylene blue, 0.05%	Diode laser, 660 nm; 180 mW; irradiation time: NA	Adjunctive PDT with PD is more effective than MD alone	Nothing to disclose
Alasqah, 2022, Saudi Arabia (38)	Peri-implantitis	BoP involving, 30% of peri-implant sites; (d) loss in supporting bone ≥3 mm around the functional implant with PPD of ≥4 mm	Control: 40.5 (30–51) Test 1: 45.4 (35–61) Test 2: 42.1 (34–55)	Non-smokers	Control: *n* = 25, MD Test 1: *n* = 23, MD + laser therapy using Er,Cr: YSGG (ECL) Test 2: MD + PDT	Methylene blue, 0.05%	Diode laser, 670 nm; 150 mW; 15 s	Photodynamic therapy and Er,Cr: YSGG adjunct to non-surgical mechanical debridement demonstrated significant improvement in peri-implant inflammatory parameters in obese individuals	Not reported
Alqutub, 2022, Saudi Arabia (39)	Peri-implant diseases	BoP on 30% of sites of peri-implant, PPD ≥4 mm/or supporting bone loss ≥3 mm	Control: 43.7 (36–60) Test 1: 43.5 (37–58) Test 2: 45.1 (34–63)	Non-smokers	Control: *n* = 32, MD with curettes Test 1: *n* = 33, MD with curettes and adjunctive PDT Test 2: *n* = 30, MD with curettes and Er,Cr:YSGG	Methylene blue, 0.005%	Diode laser, 680 nm; 150 mW; 60 s	MD with adjunctive PDT is more efficient in reducing peri-implant soft tissue inflammatory parameters than MD alone	Nothing to disclose
Shetty et al., 2022, Saudi Arabia (54)	Peri-implant mucositis	2017 new classification scheme for periodontal and peri-implant diseases and conditions	Control: 45.1 ± 3.3 Test: 42.5 ± 6.4	Non-smokers	Control: full mouth scaling and root planning, MD Test: full mouth scaling and root planning, MD with adjunctive PDT	Methylene blue, 0.005%	Diode laser, 660 nm; 150 mW; 60 s	MD with adjunctive PDT is more effective in reducing peri-implant soft tissue inflammation than MD alone	Nothing to disclose
Pourabbas et al., 2023, Iran (57)	Peri-implant mucositis	Pocket probing depth of 4–6 mm in association with bleeding on probing at ≥1 peri-implant site and radiographic evidence of bone loss with a range of 0.5‒2 mm from the time when the prosthetic reconstruction was delivered to prescreening appointment	37.5 (26–58)		Control: *n* = 24, MD Test: *n* = 25, MD with aPDT	Indocyanine green	Diode laser, 805 nm; 0.5 W; 120 s	The application of PDT using 805 nm laser and indocyanine green as an adjunct therapy to MD did not provide any additional improvements in the clinical or biologic parameters of peri-implant mucosal inflammation	Nothing to disclose
Elsadek, 2023, Saudi Arabia (56)		2017 new classification scheme for periodontal and peri-implant diseases and conditions	Control: 48.2 ± 7.8, Test 1:45.3 ± 3.9, Test 2: 47.6 ± 6.5		Control: *n* = 13, MD Test 1: *n* = 13, MD with PDT (indocyanine green) Test 2: *n* = 12, MD with PDT (methylthionine chloride)	Indocyanine green/methylene blue	Diode laser, 810 nm; 300 mW; 60 s/diode laser, 660 nm; 100 mW; 120 s	In diabetes mellitus patients with peri-implantitis, adjunctive Fox Green PDT and methylthionine chloride PDT exhibited comparable outcomes in terms of peri-implant clinical as well as pro-inflammatory characteristics than MD alone	Not reported

For the quantitative analysis, eleven studies investigated patients with peri-implantitis (43–45, 47, 49–53, 55, 56), while five studies identified patients with peri-implant mucositis (46, 48, 54, 57, 58). MD performed in two studies was surgical debridement (44, 55), while in all of the other included studies, MD was non-surgical debridement.

Subgroup analysis based on smoking habits, case definition, and photosensitisers was conducted. Photosensitisers reported in the studies include toluidine blue, methylene blue, phenothiazine chloride, and indocyanine green solution. The light source type used in all studies was a diode laser with wavelengths ranging from 635 to 810 nm while irradiation timings ranged from 10 to 120 s. For the treatment procedure, the intervention in one study was MD with multiple sessions of PDT (52). Interventions in all other included studies were MD with a single session of PDT.

Four studies only include non-smokers (45, 47, 53, 54). Two studies only (46, 58) analysed smoking patients. One study (49) divided patients into both smokers and non-smokers, and another study investigated vaping patients (48). The remaining studies did not provide a clear definition of the smoking habits of the participants.

In addition, the case definitions employed in the included studies exhibited variations, leading to distinct disease severity in the inclusion criteria among these studies. Five studies enrolled patients with PPD of at least 4 mm (42, 43, 46, 49), three studies included patients with PPD of ≥5 mm (44, 47, 55), two studies recruited patients with PPD ≥6 mm (50–52), and two studies recruited patients with PPD ranging from 4 to 6 mm (45, 57). Another three studies employed the case definition from the 2017 Classification of Periodontal and Peri-implant Diseases and Conditions (53, 54, 56). One study defined an average bone loss of 3 mm compared with baseline (41). Two studies omitted the PPD from their case definitions and inclusion criteria (48, 58).

Regarding systemic conditions, two studies specifically recruited participants with diabetes (51, 52), while another study included individuals with prediabetes (42). No other systemic diseases were involved in any of the included studies.

### 3.2 Risk of bias

Among the 26 trials, 6 were categorised as low risk of bias, 9 were with some concern, and 11 were at high risk of bias (Appendix Figure A1). Due to the limited number of studies available, it was not appropriate to assess publication bias.

### 3.3 Results of the clinical measurements

#### 3.3.1 Probing pocket depth (PPD)

**Figure 2:**
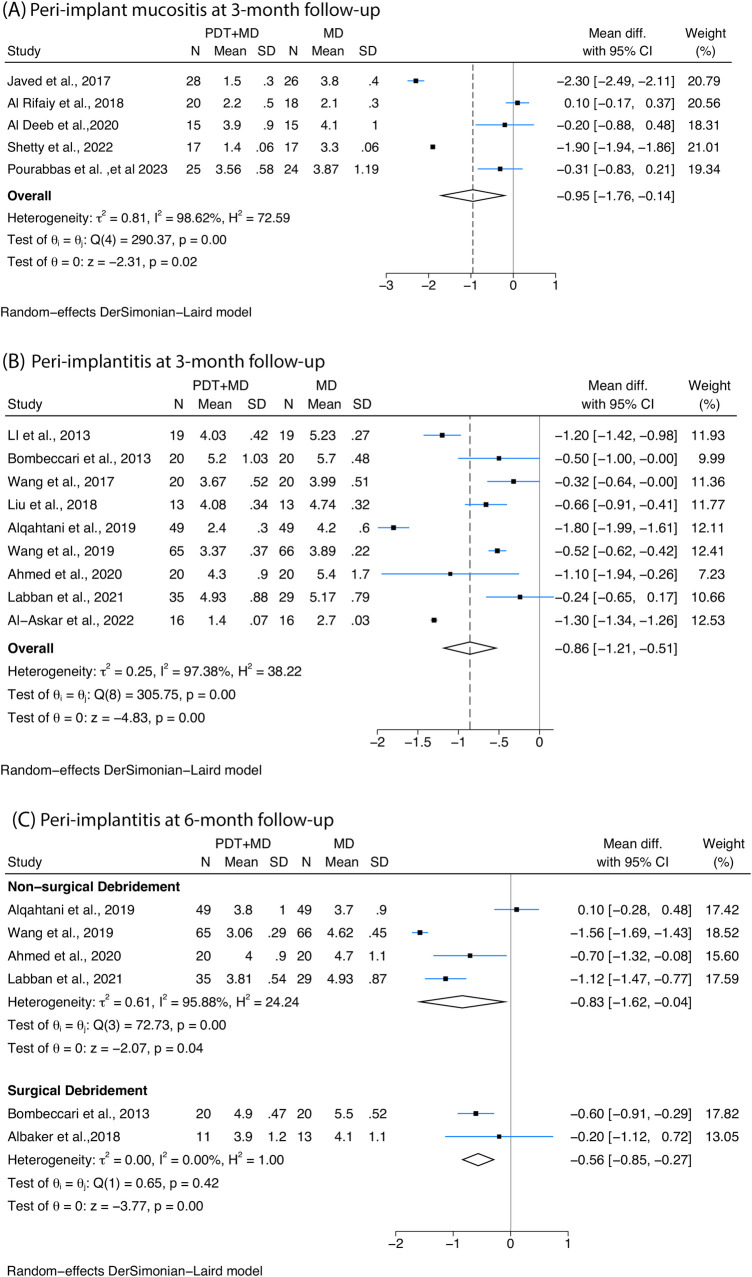
Forest plots illustrating the mean difference with 95% confidence interval (CI) in probing pocket depth (PPD) reduction posttreatment between photodynamic therapy (PDT) combined with mechanical debridement (MD) vs. MD alone. Patients receiving PDT + MD demonstrated a greater reduction in PPD than those receiving MD alone **(A)** at 3 months among peri-implant mucositis patients treated with non-surgical therapy; **(B)** at 3 months among peri-implantitis patients treated with non-surgical therapy; and **(C)** at 6 months among peri-implantitis patients receiving PDT combined with non-surgical or surgical treatment.

A greater reduction in PPD was detected after MD combined with PDT when compared with MD alone at 3-month follow-up with non-surgical therapy. Five studies with peri-implant mucositis patients confirmed a reduction of 0.95 mm [95% confidence interval (CI): −1.76 to −0.14, *I*^2^ = 98.62%, Figure 2A], and four out of five studies were using methylene blue as the photosensitiser. In addition, a mean difference of −0.86 mm (95% CI: −1.21 to −0.51, *I*^2^ = 97.38%) was observed among peri-implantitis patients (Figure 2B), five out of the nine studies used toluidine blue as the photosensitiser, while two studies used methylene blue.

Six studies with peri-implantitis assessed PPD at 6-month follow-up. Among them, four studies investigating non-surgical therapy confirmed a greater reduction of −0.83 mm (95% CI: −1.62 to −0.04, *I*^2^ = 95.88%) in patients who underwent PDT combined protocol compared with control, and four different kind of photosensitisers were employed (phenothiazine chloride, indocyanine green, methylene blue, toluidine blue). Two studies with different photosensitisers (methylene blue, toluidine blue) underwent surgical MD and also showed a significant difference of −0.56 mm (95% CI: −0.85 to −0.27, *I*^2^ = 0%) (Figure 2C) in PDT combined with MD compared with MD alone.

##### 3.3.1.1 Subgroup analyses based on case definition for non-surgical mechanical debridement

**Figure 3:**
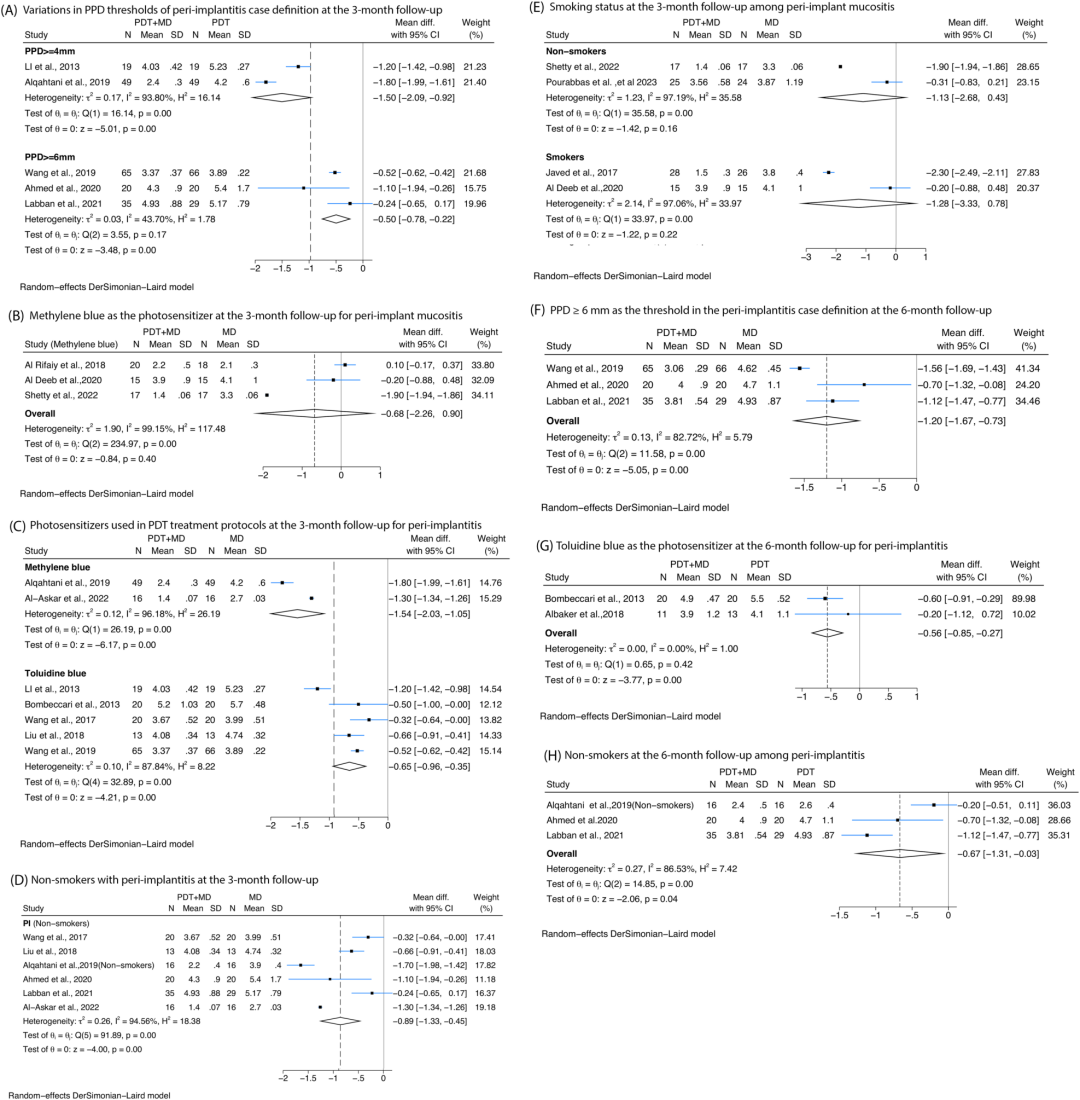
Forest plots presenting the posttreatment differences in probing pocket depth (PPD) between photodynamic therapy combined with mechanical debridement (PDT + MD) vs. mechanical debridement (MD) alone, categorised by specific variables at 3- and/or 6-month follow-ups. **(A)** Variations in PPD thresholds used in the peri-implantitis case definition at the 3-month follow-up. **(B)** Methylene blue as the photosensitiser in PDT treatment protocols at the 3-month follow-up for peri-implant mucositis. **(C)** Photosensitisers used in PDT treatment protocols at the 3-month follow-up for peri-implantitis. **(D)** Non-smokers at the 3-month follow-up among peri-implantitis patients. **(E)** Smoking status at the 3-month follow-up among peri-implant mucositis patients. **(F)** PPD ≥6 mm as the threshold in the peri-implantitis case definition at the 6-month follow-up. **(G)** Toluidine blue is the photosensitiser used in PDT protocols at the 6-month follow-up for peri-implantitis. **(H)** Non-smokers at the 6-month follow-up among peri-implantitis patients.

Firstly, at 3-month follow-up, subgroup analyses based on different PPD thresholds used in the case definition of peri-implantitis, including PPD ≥4 or 6 mm, all confirmed significant differences between PDT combined with MD compared with MD alone. For PPD ≥4 mm, a reduction of −1.5 mm (95% CI: −2.09 to −0.92, *I*^2^ = 93.8%) was observed. For PPD ≥6 mm, a reduction of −0.5 mm (95% CI: −0.78 to −0.22, *I*^2^ = 43.7%) was observed (Figure 3A). Secondly when evaluating studies of peri-implantitis with 6-month follow-up, subgroup analyses based on PPD threshold of PPD ≥6 mm showed greater reduction of −1.2 mm (95% CI: −1.67 to −0.73, *I*^2^ = 82.72%) in PPD with the treatment of MD combined with PDT compared with MD alone (Figure 3F).

##### 3.3.1.2 Subgroup analyses based on the treatment protocol of PDT for non-surgical mechanical debridement

Four types of photosensitisers at 3-month follow-up were evaluated. Toluidine blue showed no significant reductions in PPD among peri-implant mucositis patients (Figure 3B). In peri-implantitis patients, methylene blue showed a reduction of −1.54 mm (95% CI: −2.03 to −1.05, *I*^2^ = 99.18%) and then toluidine blue with a reduction of −0.65 mm (95% CI: −0.96 to −0.35, *I*^2^ = 87.84%). At 6-month follow-up, toluidine blue showed a significant reduction of −0.56 mm (95% CI: −0.85 to −0.27, *I*^2^ = 0%) (Figure 3G).

##### 3.3.1.3 Subgroup analyses based on smoking habit for non-surgical mechanical debridement

A statistically significant reduction in PPD was observed in non-smoking peri-implantitis patients treated with MD and PDT compared with MD alone at the 3-month follow-up, with a mean difference of −0.89 mm (95% CI: −1.33 to −0.45, *I*^2^ = 94.56%) (Figure 3C). However, no significant reduction in PPD was found among peri-implant mucositis patients, irrespective of smoking status (Figure 3E). Additionally, three studies evaluating non-smoking peri-implantitis patients at the 6-month follow-up reported greater PPD reductions following PDT (−0.67 mm, 95% CI: −1.31 to −0.03, *I*^2^ = 86.53%) (Figure 3H).

#### 3.3.2 Bleeding on probing (BoP)

**Figure 4:**
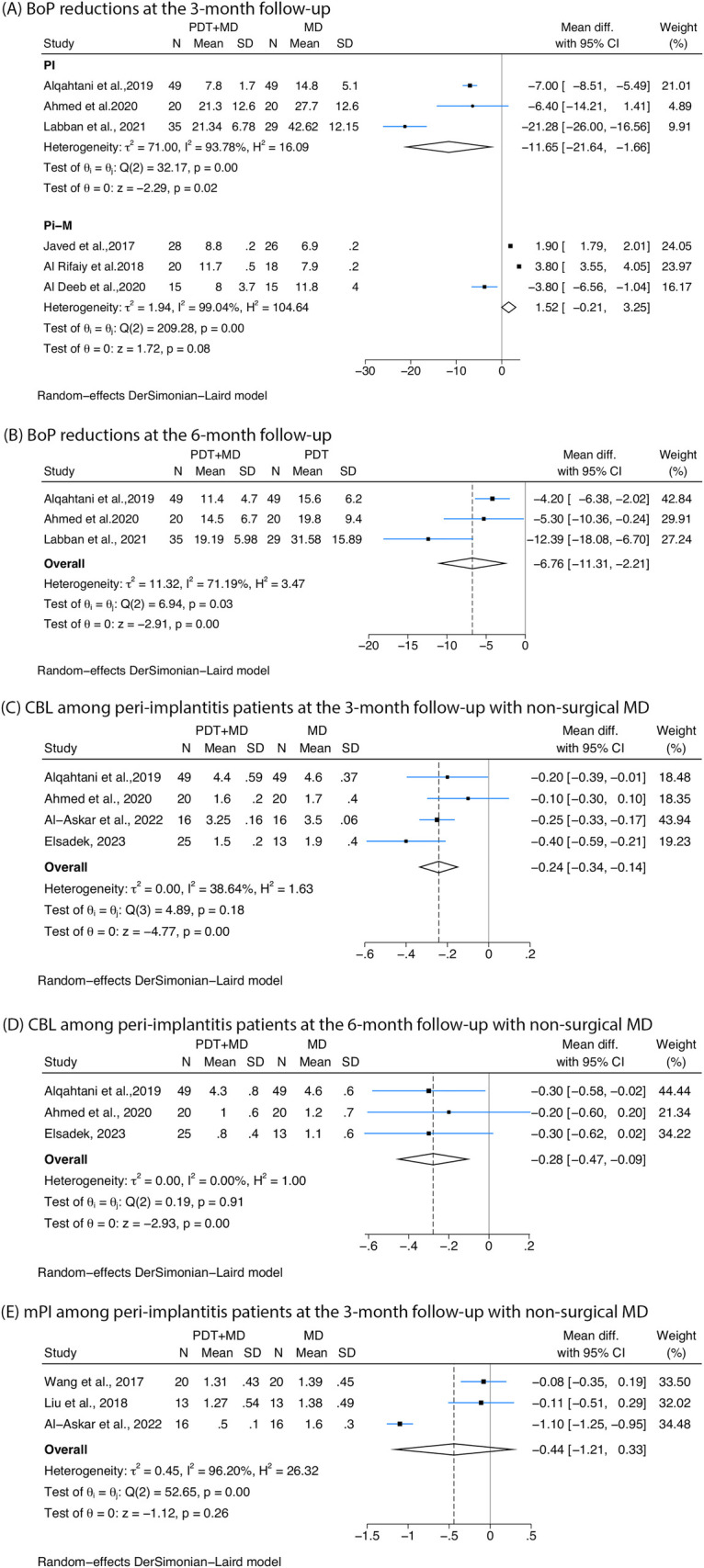
Forest plots showing the reduction in bleeding on probing (BoP), crestal bone loss (CBL), and modified plaque index (mPI) following treatment with photodynamic therapy combined with mechanical debridement (PDT + MD) vs. mechanical debridement (MD) alone. **(A)** BoP reductions in peri-implantitis (PI) and peri-implant mucositis (Pi-M) at the 3-month follow-up. **(B)** BoP reductions in peri-implantitis at the 6-month follow-up. **(C)** CBL among peri-implantitis patients at the 3-month follow-up with non-surgical MD. **(D)** CBL among peri-implantitis patients at the 6-month follow-up with non-surgical MD. **(E)** mPI among peri-implantitis patients at the 3-month follow-up with non-surgical MD.

All the studies that measured BoP used non-surgical therapy. Reductions in BoP of cases with peri-implantitis were detected at 3-month follow-up (−11.65%, 95% CI: −21.64% to −1.66%, *I*^2^ = 93.78%), while studies on peri-implant mucositis confirmed no significant difference in BoP at 3-month follow-up (Figure 4A). For peri-implantitis at 6 months, a significant reduction of −6.76% (95% CI: −11.31% to −2.21%, *I*^2^ = 71.19%) was observed (Figure 4B).

#### 3.3.3 Crestal bone loss (CBL)

Four studies reported greater reductions in CBL at 3 months among peri-implantitis cases treated with non-surgical debridement combined with PDT compared with controls (−0.24, 95% CI: −0.34 to −0.14, *I*^2^ = 38.64%) (Figure 4C). In two studies (53, 56), CBL was defined as the linear distance from 2 mm below the implant-abutment interface to the most crestal point of the adjacent alveolar bone. However, the remaining two studies (49, 51) did not clearly specify the starting or ending points used for the measurement, which may compromise the accuracy and comparability of the CBL measurements across studies. Three studies investigating peri-implantitis treated with non-surgical therapy measured CBL at 6 months, and a greater reduction was found in patients undergoing PDT (−0.28, 95% CI: −0.47 to −0.09, *I*^2^ = 0%) (Figure 4D).

#### 3.3.4 Modified plaque index (mPI)

Three studies on non-surgical therapy for peri-implantitis reported mPI at 3 months, showing no significant improvement in mPI when PDT was combined with MD compared with MD alone (Figure 4E).

### 3.4 Grading of available evidence

Although all the included studies were randomised controlled trials, resulting in a high category of the GRADE system, the overall certainty of evidence for each outcome was rated as moderate due to concerns about inconsistency. For peri-implant mucositis at 3-month follow-up, serious inconsistency was noted (*I*^2^ = 88.62%). Similarly, for peri-implantitis at 3-month follow-up, the evidence was downgraded due to high heterogeneity (*I*^2^ = 87.37%). In the case of non-surgical treatment for peri-implantitis with a 6-month follow-up, moderate certainty was assigned as well, with inconsistency explained after subgroup analysis (*I*^2^ = 85.68%). For surgical therapy at 6-month follow-up, the certainty of evidence remained moderate. These moderate-certainty ratings suggest that adjunctive PDT may be beneficial, although some caution is warranted when interpreting the results due to variability and potential reporting bias (Appendix Figure A2).

## 4 Discussion

This review indicated that PDT with MD is effective in producing greater reductions of PPD in both peri-implantitis and peri-implant mucositis, which showed a promising antibacterial efficiency. PDT with MD was superior to MD alone with regards to BoP and CBL reduction in peri-implantitis patients. However, PDT showed no significant improvement in BoP reduction among peri-implant mucositis. PDT combined with surgical MD also demonstrated better outcomes compared with surgical MD alone. However, the reduction in PPD was less pronounced than that observed with non-surgical MD, likely due to the inherently greater effectiveness of surgical MD over non-surgical approaches.

Inconclusive evidence on the topic was published before. Indeed, a previous systematic review demonstrated a reduction in both CBL and BoP at 6-month follow-up when PDT was combined with MD in patients with diabetes, as compared with MD alone. However, no substantial changes in PPD were observed (59). A similar review involved patients with peri-implant mucositis, confirming improvements in PPD when PDT was combined with MD but differences in BoP (60). When analysing the impact of treatment in patients who smoked and had peri-implant diseases, the combination of MD and PDT resulted in a greater reduction in both PPD and PI (61) than the control.

A previous study indicated that increased PPD is associated with a pathogenic bacterial boost (62). Moreover, a systematic review demonstrated that heterogeneous mixed infection was detected around inflamed implants with predominantly non-culturable Gram-negative species compared with periodontitis (63). PDT has been suggested as an alternative therapy for peri-implant diseases in eliminating *Aggregatibacter actinomycetemcomitans*, *Porphyromonas gingivalis*, and *Prevotella intermedia* (64). Gram-positive species, including *staphylococci*, were also detected (6). Gram-positive bacteria are more susceptible to anionic and neutral photosensitisers due to their porous cell wall, while Gram-negative bacteria are more resistant because of their outer membrane (65). However, cationic photosensitisers are effective against both types, showing enhanced phototoxicity in various studies (66, 67).

An increasing number of studies have focused on the use of systemically administered antimicrobials for managing peri-implantitis; however, the findings on their antimicrobial effectiveness have been inconsistent. Two studies have shown improvements in peri-implantitis in patients who received systemic antibiotics, including amoxicillin, azithromycin, and metronidazole combined with non-surgical treatment (68, 69). But this approach seemed to have failed to reduce the need for additional surgical therapy (70, 71). In addition, a study revealed that only 50% of the cases with systemic antibiotics combined with surgical treatment showed improvement after 12 months (72). Given the risk of antibiotic resistance and the uncertain effectiveness of antibiotics in treating peri-implantitis, caution should be exercised in their administration. Overuse or unnecessary use of antibiotics can contribute to the development of resistant bacterial strains, posing a potential risk to public health (72). Unlike antibiotics, PDT does not pose a risk of resistance and can be administered without dose limitations, making it unlikely for resistance to develop even with repeated treatments. PDT also exhibits several additional advantages. It is straightforward to use in clinical settings as a portable device, and the procedure typically lasts only a few minutes. With this, PDT treatment is considered relatively cost-effective compared with other approaches, such as surgical debridement. Given its demonstrated effectiveness when combined with non-surgical therapy, PDT may reduce the need for more invasive surgical interventions, thereby potentially lowering the overall cost of managing peri-implant disease. However, current evidence comparing the cost of PDT with other adjunctive treatments remains limited, and further economic evaluations are needed to substantiate its cost-effectiveness.

This review poses an important question of whether it should be used more, combined with MD protocols for controlling bacteria, to reduce the need for surgical access procedures.

The long-term efficiency of PDT should be explored in future studies. One randomised clinical study that evaluated PPD at 1-year follow-up revealed that no additional benefits were observed in the PDT group, and recurrence of peri-implantitis affected groups was found either with or without PDT (41). Another study from Javed et al. (35) demonstrated no significant difference at 12-month follow-up of PPD, also providing evidence for the hypothesis. These findings may be attributed to several factors. First, the antimicrobial effects of PDT are transient, and without regular maintenance therapy or repeated PDT sessions, the clinical benefits may not be sustained over time due to its limited capacity to fully eliminate the complex biofilms on implant surfaces (35). Secondly, host factors, such as the patient's immune response and systemic health status, may influence the long-term response to therapy (73).

Smoking habit is considered a factor impacting the efficacy of PDT. Previously conducted reviews illustrated the efficacy of PDT in the treatment of peri-implant diseases among smokers and indicated that PDT improves the condition of peri-implant diseases even if excluding non-smoking patients (61, 74). Other studies also documented that tobacco use contributes to a poor response to supportive periodontal treatment (75, 76). However, it is noteworthy that smoking exerts a continuous and strong suppressive effect on gingival bleeding (77). Therefore, caution should be taken in the analysis of BoP, since the efficacy of PDT on smokers can be masked with regard to BoP assessment. Accordingly, more studies should be conducted to explore whether smoking could reduce the efficacy of PDT on peri-implant diseases.

A difference between PDT treatment protocols was noted in this review. Although all the test groups of the trials included in this review adopted protocols of MD with PDT, several photosensitisers (including toluidine blue, methylene blue, phenothiazine chloride, and indocyanine green solution), different illumination times, and various wavelengths were utilised. The antimicrobial efficacy is closely related to the photosensitisers, and an appropriate light source must be matched to the delivery device and photosensitiser to achieve the most effective outcome (78). This review revealed a decreasing efficacy in reducing PPD at the 3-month follow-up among peri-implantitis, with the order being methylene blue and then toluidine blue. However, it is important to interpret these results with caution, given the limited number of studies available; further research should be conducted to investigate which photosensitiser and which light wavelength yield the highest effectiveness in PDT. Moreover, there is a requirement to develop a specific protocol for applying PDT in the treatment of peri-implant diseases.

Another contributing factor to the heterogeneity is the variability in case definitions across the included studies. Most of these studies mentioned PPD in their case definitions; lower heterogeneity was observed when categorising the studies based on different PPD thresholds. Therefore, standardising case definitions is crucial to ensure more accurate and consistent results. It is highly recommended to adopt the case definition outlined in the 2017 World Workshop on the Classification of Periodontal and Peri-implant Diseases and Conditions (2) in future studies.

This review has several limitations that should be considered. Firstly, a moderate-to-high risk of bias was observed in a substantial number of clinical trials based on the RoB 2 assessment tool. When considered alongside the GRADE evaluation, which rated the certainty of evidence as moderate primarily due to inconsistency, it becomes evident that the strength and reliability of the conclusions are limited. Although the available evidence suggests a potential benefit of PDT as an adjunctive therapy, these methodological concerns highlight the need for more robust, low-risk studies to confirm its efficacy. Secondly, the meta-analysis included studies with a maximum of 3- and 6-month follow-ups; hence, it is unclear whether the greater reductions observed with PDT are sustained beyond these short-term periods. Thirdly, high levels of heterogeneity of the results were reported, which appeared to be influenced by factors such as smoking status, case definitions, and variations in the protocols used for the application of photosensitisers. However, the possibility of additional unassessed confounding factors cannot be excluded. Additionally, the inclusion of patients with diabetes in the analysed studies may have impacted treatment efficacy, as diabetes is known to impair healing and modulate inflammatory responses. This could have introduced variability in the observed outcomes and potentially affected the accuracy of efficacy assessments. On the other hand, the strengths of this systematic review should be considered. The approach in reviewing this topic followed rigorous methods following a preregistered protocol, with a well-defined methodology and process. Confounding factors were considered, and subgroup analyses were developed to explore the reason of high heterogeneity. Lower levels of heterogeneity were detected after the subgroup analysis based on the PPD thresholds defined in the study case definitions and based on different photosensitisers.

## 5 Conclusion

This review emphasises the effectiveness of PDT in managing peri-implant diseases. When combined with MD, PDT led to greater reductions in PPD and CBL, along with significant improvements in BoP. PDT seems to be an accessible, low-cost strategy to target polymicrobial communities without contributing to antimicrobial resistance, making it a promising adjunctive treatment that can be widely implemented by trained dental professionals. However, these conclusions should be interpreted with caution due to the moderate certainty of evidence rated by the GRADE approach and the moderate to high risk of bias observed in several included studies. Moreover, the long-term effects of PDT on the microbial composition of peri-implant biofilms remain underexplored. Although this review highlights the antimicrobial potential of PDT, the included studies did not directly evaluate the decontaminating effects. Future research is needed to clarify the impact of PDT on microbial ecology over time, standardise treatment protocols, and explore potential synergistic effects with other antimicrobial strategies through well-designed, low-bias clinical trials.

## Data Availability

The original contributions presented in the study are included in the article/Supplementary Material; further inquiries can be directed to the corresponding author.
