# Supplementary material for: Photodynamic therapy and peri-implant diseases: a systematic review and meta-analysis
Source: Front Oral Health. 2025 Jul 9;6:1614982. doi: 10.3389/froh.2025.1614982 (PMC12283991; doi:10.3389/froh.2025.1614982)
Supplement: Supplementary file 1 [file Table1.docx]

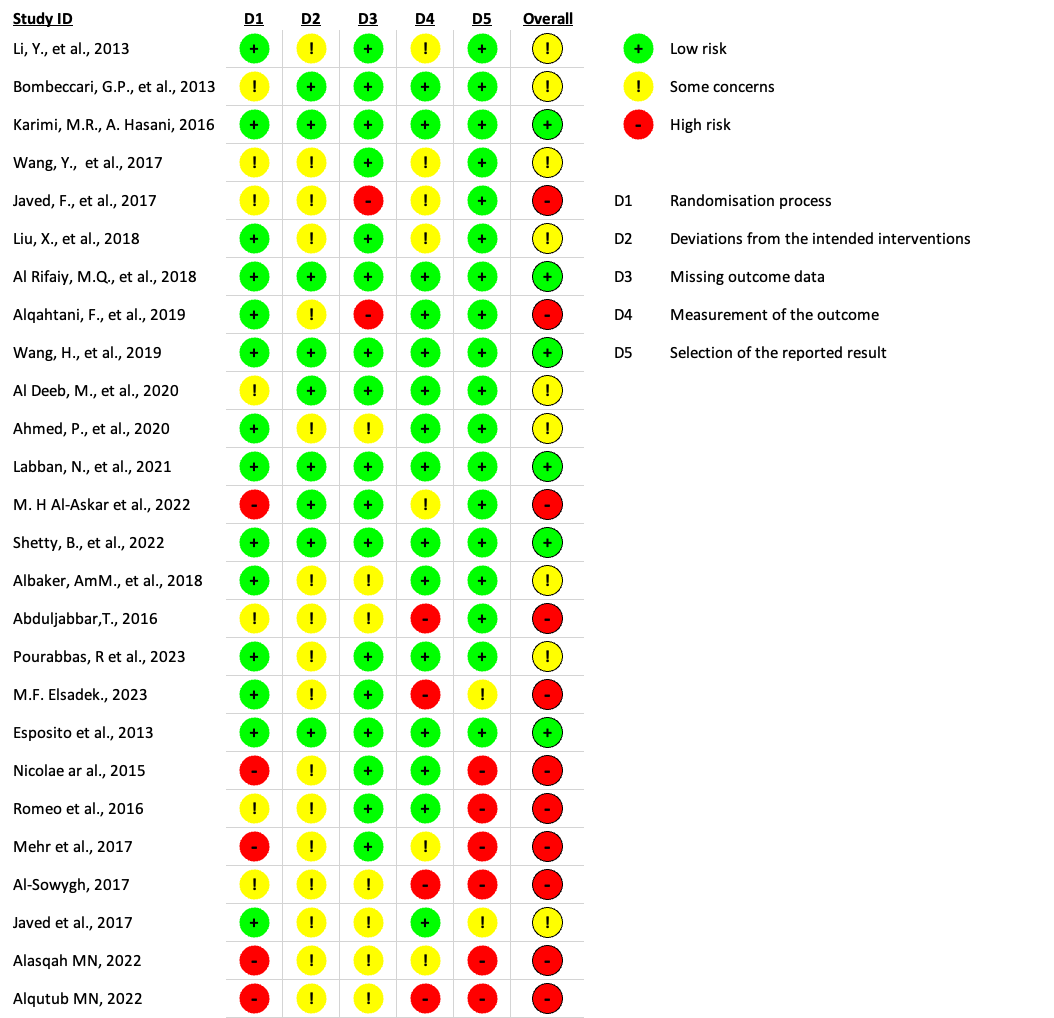


Appendix FIGURE 1

Risk of bias assessed by ROB2 TOOL. In detail, 6 of 26 trials were at low risk of bias, 9 exhibited some concern, whilst 11 were at high risk of bias.


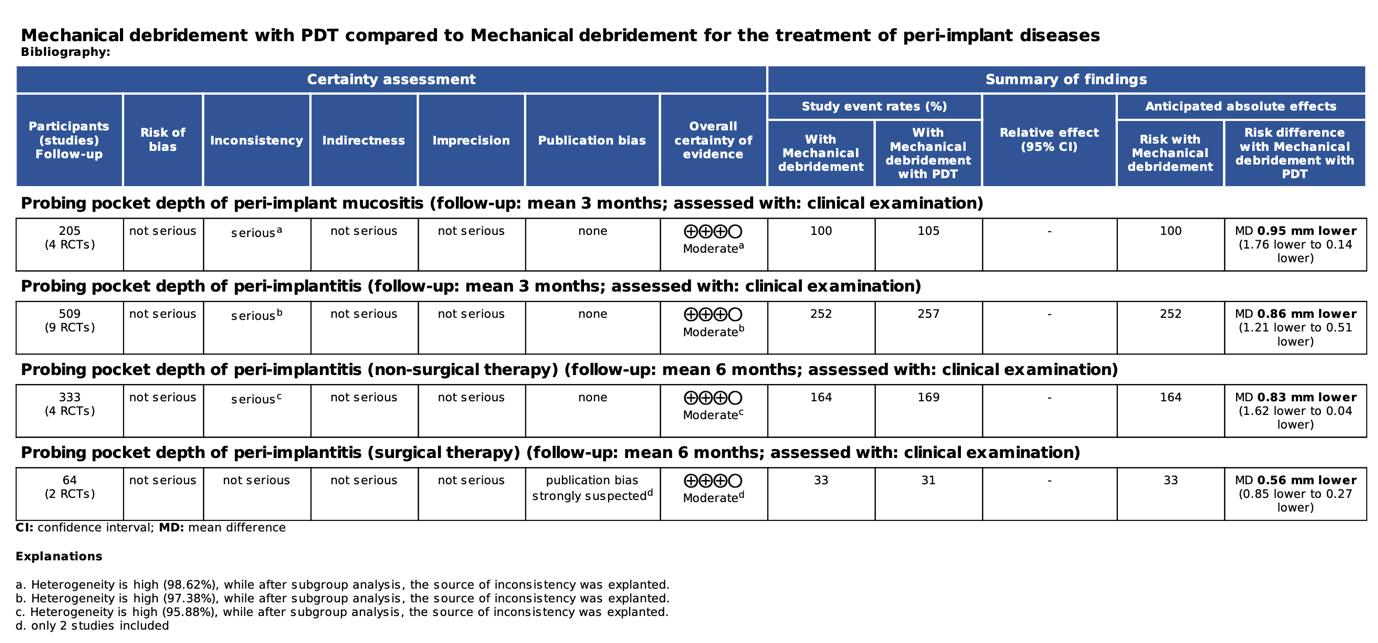


Appendix FIGURE 2

GRADE (Grading of Recommendations Assessment, Development and Evaluation) system for assessing the quality of evidence in studies
