# Supplementary material for: Photodynamic therapy and peri-implant diseases: a systematic review and meta-analysis
Source: Front Oral Health. 2025 Jul 9;6:1614982. doi: 10.3389/froh.2025.1614982 (PMC12283991; doi:10.3389/froh.2025.1614982)
Supplement: Supplementary file 2 [file Table2.docx]

| The aspect of the research question in terms of outcomes that could be of interest:  evidence of the efficacy of PDT on peri-implant diseases. | |
| --- | --- |
| **1.1       Search strategy via Ovid MEDLINE(R)** | |
| 1 | Peri-Implantitis/ |
| 2 | peri-implant disease*.mp. |
| 3 | peri-implant mucositis.mp. |
| 4 | periimplant mucositis.mp. |
| 5 | Peri-Implantitides.mp. |
| 6 | dental implant* inflammation.mp. |
| 7 | dental implant* bone loss.mp. |
| 8 | inflammation of the dental implants.mp. |
| 9 | (inflam* adj3 dental implant*).mp. |
| 10 | periimplantitides.mp. |
| 11 | 1 or 2 or 3 or 4 or 5 or 6 or 7 or 8 or 9 or 10 |
| 12 | Photochemotherapy/ |
| 13 | Photochemotherap*.mp. |
| 14 | Photo-chemotherap*.mp. |
| 15 | PDT.mp. |
| 16 | Photodynamic Therap*.mp. |
| 17 | Photo-dynamic Therap*.mp. |
| 18 | la?er*.mp. |
| 19 | 12 or 13 or 14 or 15 or 16 or 17 or 18 |
| 20 | 11 and 19 |
|  |  |
| **1.2       Search Strategy via Ovid Embase** | |
| 1 | Peri-Implantitis/ |
| 2 | peri-implant disease*.mp. |
| 3 | peri-implant mucositis.mp. |
| 4 | periimplant mucositis.mp. |
| 5 | peri-implantitides.mp. |
| 6 | periimplantitides.mp. |
| 7 | dental implant* inflammation.mp. |
| 8 | dental implant* bone loss.mp. |
| 9 | inflammation of the dental implants.mp. |
| 10 | (inflam* adj3 dental implant*).mp.. |
| 11 | 1 or 2 or 3 or 4 or 5 or 6 or 7 or 8 or 9 or 10 |
| 12 | Photochemotherapy/ |
| 13 | Photochemotherap*.mp. |
| 14 | Photo-chemotherap*.mp. |
| 15 | PDT.mp. |
| 16 | Photodynamic Therap*.mp. |
| 17 | Photo-dynamic Therap*.mp. |
| 18 | la?er*.mp. |
| 19 | 12 or 13 or 14 or 15 or 16 or 17 or 18 |
| 20 | 11 and 19 |
|  |  |
| **1.3       Search Strategy via Cochrane Central Register for Controlled Trials (CENTRAL)** | |
| #1 | MeSH descriptor: [Peri-Implantitis] explode all trees |
| #2 | (peri-implant disease*):ti,ab,kw |
| #3 | (peri-implant mucositis):ti,ab,kw |
| #4 | (periimplant mucositis):ti,ab,kw |
| #5 | (peri-implantitides):ti,ab,kw |
| #6 | (periimplantitides):ti,ab,kw |
| #7 | (dental implant* inflammation):ti,ab,kw |
| #8 | (dental implant* bone loss):ti,ab,kw |
| #9 | (inflammation of the dental implants):ti,ab,kw |
| #10 | (inflam* near/3 dental implant*):ti,ab,kw |
| #11 | MeSH descriptor: [Photochemotherapy] explode all trees |
| #12 | (Photochemotherap*):ti,ab,kw |
| #13 | (Photo-chemotherap*):ti,ab,kw |
| #14 | (PDT.mp.):ti,ab,kw |
| #15 | (Photodynamic Therap*):ti,ab,kw |
| #16 | (Photo-dynamic Therap*):ti,ab,kw |
| #17 | (la?er*):ti,ab,kw |
| #18 | #1 or #2 or #3 or #4 or #5 or #6 or #7 or #8 or #9 or #10 |
| #19 | #11 or #12 or #13 or #14 or #15 or #16 or #17 |
| #20 | #18 and #19 |
|  |  |
| **1.4       Search Strategy for Web of Science** | |
| #1. TS=(Peri-Implantitis OR "peri-implant disease*" OR "peri-implant mucositis" OR "periimplant mucositis" OR peri-implantitides OR Periimplantitides OR "dental implant* inflammation" OR "dental implant* bone loss" OR "inflammation of the dental implants" OR inflam* NEAR/4 "dental implant*") | |
| #2.TS=(Photochemotherapy OR Photochemotherap* OR Photochemotherap* OR PDT OR "Photodynamic Therap*" OR Photo-dynamic Therap* OR laser* OR lazer*) | |
| #3. #2 AND #1 | |
|  |  |
| **1.5       Search Strategy via Dentistry & Oral Sciences Source** | |
| S1. TI "peri-implant disease*" OR AB "peri-implant disease*" OR KW "peri-implant disease*" | |
| S2. TI "peri-implant mucositis" OR AB "peri-implant mucositis" OR KW "peri-implant mucositis" | |
| S3. DE "Peri-Implantitis" | |
| S4. TI "periimplant mucositis" OR AB "periimplant mucositis" OR KW "periimplant mucositis" | |
| S5. TI "dental implant* inflammation" OR AB "dental implant* inflammation" OR KW "dental implant* inflammation" | |
| S6. TI "dental implant* bone loss" OR AB "dental implant* bone loss" OR KW "dental implant* bone loss" | |
| S7. TI "inflammation of the dental implants" OR AB "inflammation of the dental implants" OR KW "inflammation of the dental implants" | |
| S8. TI "inflam* near/3 dental implant*" OR AB "inflam* near/3 dental implant*" OR KW "inflam* near/3 dental implant*" | |
| S9. DE Photochemotherapy | |
| S10. TI Photochemotherap* OR AB Photochemotherap* OR KW Photochemotherap* | |
| S11. TI "Photo-chemotherap*" OR AB "Photo-chemotherap*" OR KW "Photo-chemotherap*" | |
| S12. TI PDT OR AB PDT OR KW PDT | |
| S13. TI "Photodynamic Therap*" OR AB "Photodynamic Therap*" OR KW "Photodynamic Therap*" | |
| S14. TI "Photo-dynamic Therap*" OR AB "Photo-dynamic Therap*" OR KW "Photo-dynamic Therap*" | |
| S15. TI la?er*T OR AB la?er*T OR KW la?er* | |
| S16. S1 OR S2 OR S3 OR S4 OR S5 OR S6 OR S7 OR S8 | |
| S17. S9 OR S10 OR S11 OR S12 OR S13 OR S14 OR S15 | |
| S18. S16 AND S17 | |
|  |  |
| **1.6       Search strategy for Scopus** | |
| ( TITLE-ABS-KEY ( peri-implantitis  OR  "peri-implant disease*"  OR  "peri-implant mucositis"  OR  "periimplant mucositis"  OR  peri-implantitides  OR  periimplantitides  OR  "dental implant* inflammation"  OR  "dental implant* bone loss"  OR  "inflammation of the dental implants"  OR  "inflam* pre3 dental implant*" )  AND  TITLE-ABS-KEY ( photochemotherapy  OR  photochemotherap*  OR  photo-chemotherap*  OR  pdt  OR  "Photodynamic Therap*"  OR  "Photo-dynamic Therap*"  OR  la?er* ) ) | |
|  | |
| 1.7       **Search Strategy for LILACS via Virtual Health Library** | |
| (mh:(peri-implantitis) OR TW:((peri-implant disease*) OR (peri-implant mucositis) OR (periimplant mucositis) OR (Peri-Implantitides) OR ("dental implant* inflammation") OR ("dental implant* bone loss") OR ("inflammation of the dental implants") OR (periimplantitides) OR ("inflam* adj3 dental implant*")))AND (mh:(Photochemotherapy) OR tw:((Photochemotherap*) OR (Photo-chemotherap*) OR (PDT) OR (Photodynamic Therap*) OR (Photo-dynamic Therap*) OR (la?er*))) | |
|  | |
| 1.8       **Search strategy for China Online** | |
| （中英文扩展&主题词扩展）： 主题:("peri-implantitis" or "peri-implant diseases” or “peri-implant mucositis” or “periimplant mucositis” or “peri-implantitides” or “periimplantitides” or “dental implant inflammation” or “dental implant bone loss” or “inflammation of the dental implants” or “periimplantitides”) and 主题:("Photochemotherapy" or “Photochemotherapies” or “Photo-chemotherapy” or “Photo-chemotherapies” or “PDT” or “Photodynamic Therapy” or “Photo-dynamic Therapies” or “Photo-dynamic Therapy” or “Photo-dynamic Therapy” or “laser” or “lasers” or “lazer” or “lazers”) | |
|  | |
| **1.9       Hand search** | |
| The bibliographies of papers will be checked for studies not retrieved through other search methods. | |
| The following journals will be searched by hand since 2005. | |
| Journal of Periodontology | |
| Journal of Clinical Periodontology | |
| European Journal of Oral Implantology | |
| Clinical Oral Implants Research | |
| Journal of Oral Implantology | |
| Clinical Implant Dentistry and Related Research | |
| International Journal of Oral Implantology | |

Appendix Table 1: Search strategy across Cochrane Central Register for Controlled Trials (CENTRAL), MEDLINE, EMBASE, Web of Science, Dentistry & Oral Sciences Source, Scopus, LILACS, and China Online and journals that for hand search
